# Environmental Perturbations during the Rehabilitation of Wild Migratory Birds Induce Gut Microbiome Alteration and Antibiotic Resistance Acquisition

**DOI:** 10.1128/spectrum.01163-22

**Published:** 2022-06-22

**Authors:** Hyokeun Song, Saehah Yi, Woo-Hyun Kim, Jae-Ho Guk, Minjong Ha, Insik Kwak, Janghee Han, Seong-Chan Yeon, Seongbeom Cho

**Affiliations:** a College of Veterinary Medicine and Research Institute for Veterinary Science, Seoul National University, Seoul, South Korea; b Seoul Wildlife Center, Seoul, South Korea; c Center for Veterinary Integrated Medicine Research, College of Veterinary Medicine, Seoul National University, Seoul, South Korea; Texas A&M University

**Keywords:** rehabilitation, microbiota, dysbiosis, wild birds, antibiotic resistance

## Abstract

Wild migratory birds are essential for sustaining healthy ecosystems, but the effects of a rehabilitation period on their gut microbiomes are still unclear. Here, we performed longitudinal sampling, 16S rRNA sequencing, and antibiotic resistance monitoring of the gut microbiome of six species of wild migratory birds protected as natural monuments in South Korea that are subject to short- or long-term rehabilitation periods. Overall, gut microbiome diversity was significantly decreased in the early stages of rehabilitation, and it did not recover to a level comparable to that of wild birds. Moreover, while the abundance of short-chain fatty acid-producing bacteria decreased, that of zoonotic pathogens increased, indicating rehabilitation-induced dysbiosis. The metabolic pathways involved in the degradation of aromatic pollutants were significantly downregulated, suggesting the depletion of pollutant-degrading microorganisms. Antibiotic resistance of Escherichia coli significantly increased during rehabilitation, particularly ciprofloxacin and tetracycline resistance, and seven of the rehabilitated wild birds acquired multidrug resistance. The diet and habitat changes experienced by wild migratory birds during rehabilitation may have induced the observed gut microbiome dysbiosis and acquisition of antibiotic resistance. These rehabilitation-induced alterations might affect the adaptability of wild birds to their natural environments and contribute to the spread of antibiotic resistance after their release.

**IMPORTANCE** Wild migratory birds are key for ecosystem health but highly sensitive to anthropogenic activities. Therefore, wild migratory birds often undergo rehabilitation to prevent species extinction or biodiversity monitoring. However, the impact of rehabilitation on the gut microbiome of wild migratory birds, which is closely associated with host fitness, remains unclear. For the migratory bird species considered natural monuments in South Korea evaluated here, such impacts could include rehabilitation-induced gut microbiome dysbiosis and acquisition of antibiotic resistance, with possible repercussions on the adaptability of wild birds and spread of antibiotic resistance in the environment after their release. Therefore, the dynamics of the gut microbiome and antibiotic resistance should be considered for implementing sustainable rehabilitation strategies.

## INTRODUCTION

Wild migratory birds play an essential role in sustaining a healthy ecosystem. For instance, wild migratory birds contribute to shaping the distribution of global biodiversity, as they are involved in the long-distance dispersal of various organisms, including seeds and microorganisms (1). Moreover, as they may frequently interact with humans, they are sensitive to human activities (2, 3). Owing to their high position in the food chain and sensitivity to both natural and anthropogenic environmental changes, wild migratory birds are recognized as highly effective indicators of biodiversity (4). However, migratory birds are facing an ever-growing anthropogenic threat due to the increasing modifications of their natural habitats and global climate change (5). Consequently, several migratory bird species have been classified as endangered by the International Union for Conservation of Nature (IUCN) or as natural monuments (5–7). Therefore, an improved understanding of the impact of human activities on wild migratory birds is required for the development of sustainable conservation strategies.

The gut microbiome is a symbiotic community of microorganisms, including bacteria, fungi, and viruses and their genomes (8–10). It is widely recognized that the gut microbiome is closely associated with host fitness, including its genetics, digestion, immune response, metabolic functions, and pathogen resistance, via complex host-microorganism interactions (11–14). The gut microbiome of birds is likely to differ from that of mammals because birds have unique digestive, reproductive, and immune systems (15). However, similar to the gut microbiome of other animals, that of birds also consists of beneficial, commensal, and pathogenic microorganisms, and it is shaped by various factors, including host genetics, diet, behavior, and environment. As most studies on the gut microbiome of wild birds have been conducted in natural populations (16–19), the effect of human activities on the dynamics of the gut microbiome of wild birds is not yet fully understood.

Wildlife rehabilitation is a human-related activity in which a variety of wild animal species are bred or grown and then reintroduced into their natural habitats; this is mostly performed to prevent species extinction and for biodiversity monitoring (20). In the rehabilitation center, the environmental factors that shape the gut microbiome of wild birds, such as diet and habitat, differ from those in the natural habitat. As the gut microbiome is associated with host fitness, changes in the gut microbiome induced during the rehabilitation process may have profound effects on the birds’ adaptability to the wild environment after they are released. Indeed, recent studies have reported a shift in the gut microbiome of wild animals due to rehabilitation (21–24). However, most studies on the effects of rehabilitation on the dynamics of the gut microbiome have been conducted on mammals and reptiles; hence, the impact of rehabilitation on wild birds remains unclear.

It is essential to improve the understanding of the dynamics of the gut microbiome of wild birds in response to rehabilitation to establish a sustainable conservation strategy for these species. Thus, in the present study, we investigated the effect of rehabilitation on the gut microbiome of wild migratory birds. We performed longitudinal sampling, 16S rRNA sequencing analysis of the gut microbiome, and antibiotic resistance monitoring on six species of wild migratory birds that are protected as natural monuments in South Korea. Individuals of Falco tinnunculus, Falco subbuteo, Otus bakkamoena, Otus scops, Ninox scutulata, and Accipiter gentilis kept in a rehabilitation center were evaluated from the wild state (immediately after rescue) to the release state (immediately before release). Our study revealed the dynamics of the gut microbiome, including changes in the taxonomic composition, diversity, bacterial network, and potential metabolic pathways, as well as changes in antibiotic resistance, which may affect host fitness after release to the natural habitat. This study provides information for developing sustainable rehabilitation strategies for wild birds.

## RESULTS

### Taxonomic composition of the gut microbiome of wild birds in wild and release states.

**FIG 1:**
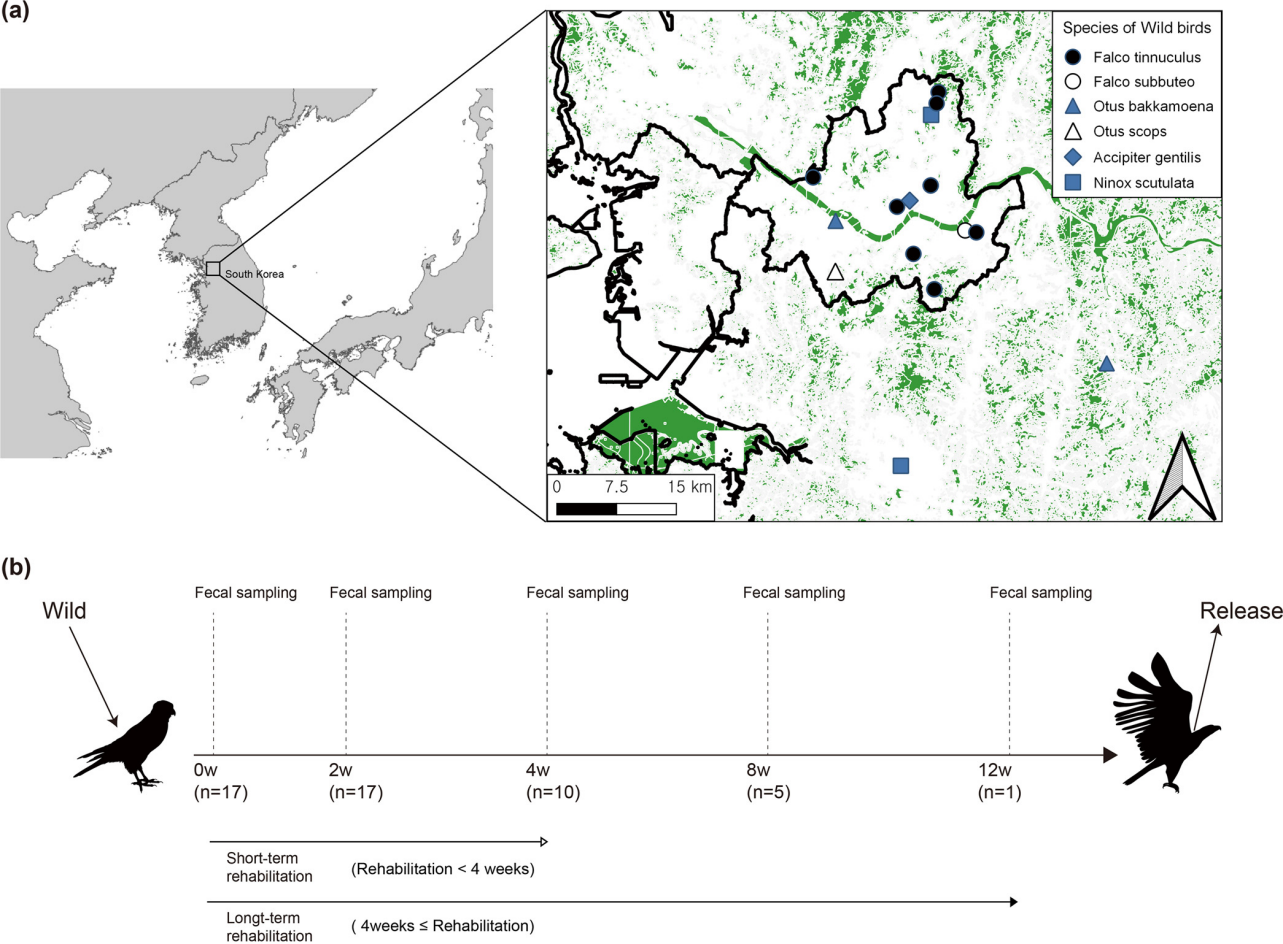
Information on wild migratory birds and the study design. (a) Rescue spots of the 17 wild migratory birds that were transferred to the Seoul Wildlife Center and used in the present study. The map was produced using the Quantum Geographical Information System version 3.16.16 (http://qgis.org) based on GPS coordinates. (b) Graphical representation of the study design and sample collection times.

**FIG 2:**
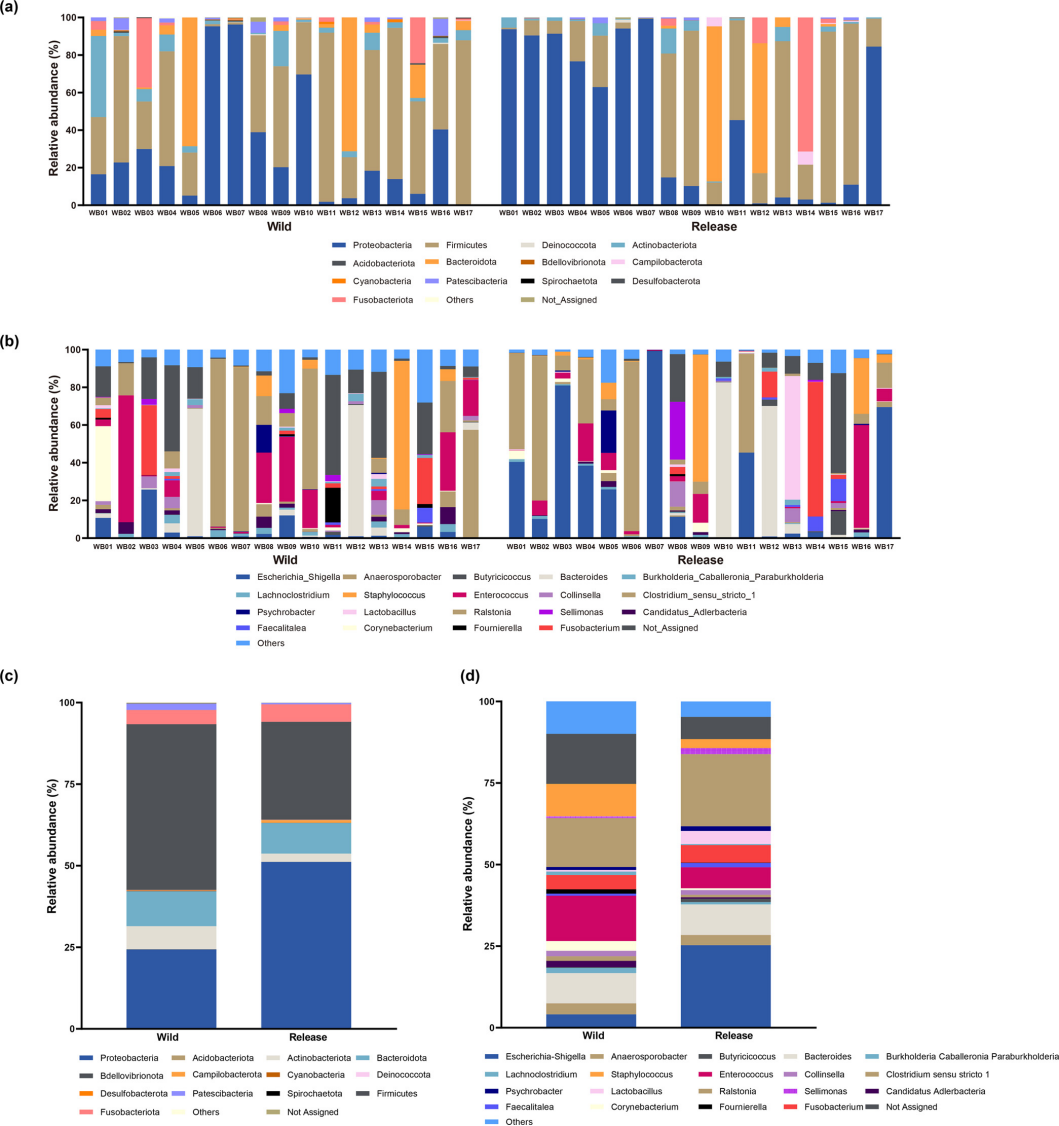
(a and b) Gut microbiome taxonomic composition in the wild and release states of the wild migratory birds at the phylum level (a) and genus level (b). Only the top 20 genera are shown. (c and d) Merged bar plots show the taxonomy composition in the wild and release states at the phylum level (c) and the genus level (d).

The overall scheme of our sampling and study design is shown Fig. 1. To understand how the gut microbiome changes during rehabilitation, we first analyzed its taxonomic composition in both the wild and release states. The taxonomic composition of the gut microbiome at the phylum and genus levels is shown in Fig. 2.

*Firmicutes*, *Proteobacteria*, *Bacteroidota*, *Actinobacteriota*, *Fusobacteriota*, and *Patescibacteria* were the six dominant phyla in both the wild and release states (accounting for 97.72 to 100% and 93.00 to 100% of total abundance, respectively). In the wild state, *Firmicutes* was the most enriched phylum across all samples (average, 50.79%), followed by *Proteobacteria* (average, 24.33%) and *Bacteroidetes* (average, 10.70%). In contrast, in the release state, *Proteobacteria* was the most enriched phylum across all samples (average, 51.16%), followed by *Firmicutes* and *Bacteroidetes* (averages of 30.00% and 9.41%, respectively).

The dominant genera differed between the wild and release states. For instance, *Ralstonia* and *Enterococcus* were the most enriched genera in the wild state (averages of 15.00% and 13.88%, respectively), followed by Staphylococcus and *Bacteroides* (averages of 9.92% and 9.26%, respectively). In the release state, Escherichia*-Shigella* and *Ralstonia* were the most enriched genera (averages of 25.27% and 21.11%, respectively), followed by *Bacteroides* and *Enterococcus* (averages of 9.40% and 6.42%, respectively).

### Rehabilitation induces the rapid and irreversible decrease of the gut microbiome alpha diversity.

**FIG 3:**
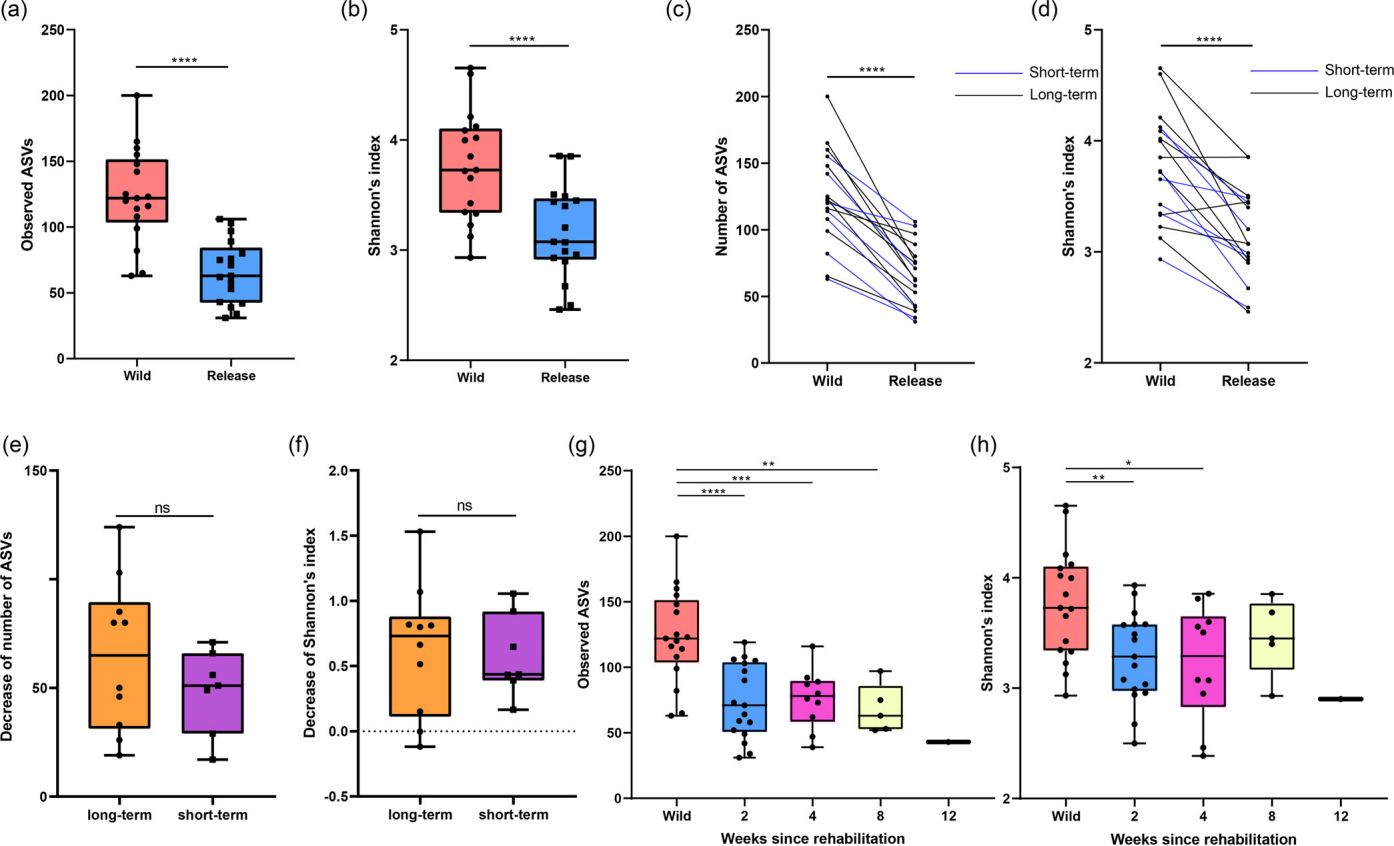
Decreased alpha diversity of the gut microbiome of wild migratory birds after short- and long-term rehabilitation. (a and b) Box plots show the decrease in the number of observed ASVs (a) and Shannon’s index (b) in the wild and release states. (c and d) Dot plots show the paired sample analysis of the number of observed ASVs (c) and Shannon’s index (d) in the wild and release states. (e and f) Box plots show the decrease in the number of ASVs (e) and Shannon’s index (f) in long-term and short-term rehabilitation groups. (g and h) Box plots show the longitudinal dynamics of the number of observed ASVs (g) and Shannon’s index (g) throughout the rehabilitation period.

We investigated the dynamics of alpha diversity during rehabilitation to determine whether dysbiosis of the gut microbiome occurred. We analyzed the shift in the alpha diversity of the gut microbiome based on two indices: the number of amplicon sequence variants (ASVs) and Shannon’s index. Both indices showed significantly lower values (*P < *0.05) in release birds than in wild birds (Fig. 3a and b). Notably, the Wilcoxon test for paired samples showed that the values of both alpha-diversity indices decreased significantly (*P < *0.05) during long- and short-term rehabilitation (Fig. 3c and d). However, the decreases in the values of both alpha diversity indices were not significantly different (*P > *0.05) between the short- and long-term rehabilitation groups (Fig. 3e and f). Longitudinal analysis revealed that the alpha diversity of the gut microbiome significantly decreased (*P < *0.05) in the first 2 weeks of rehabilitation, and it did not recover to the wild-state level during long-term rehabilitation (Fig. 3g and h).

### Shifts in the gut microbiome beta diversity during rehabilitation.

**FIG 4:**
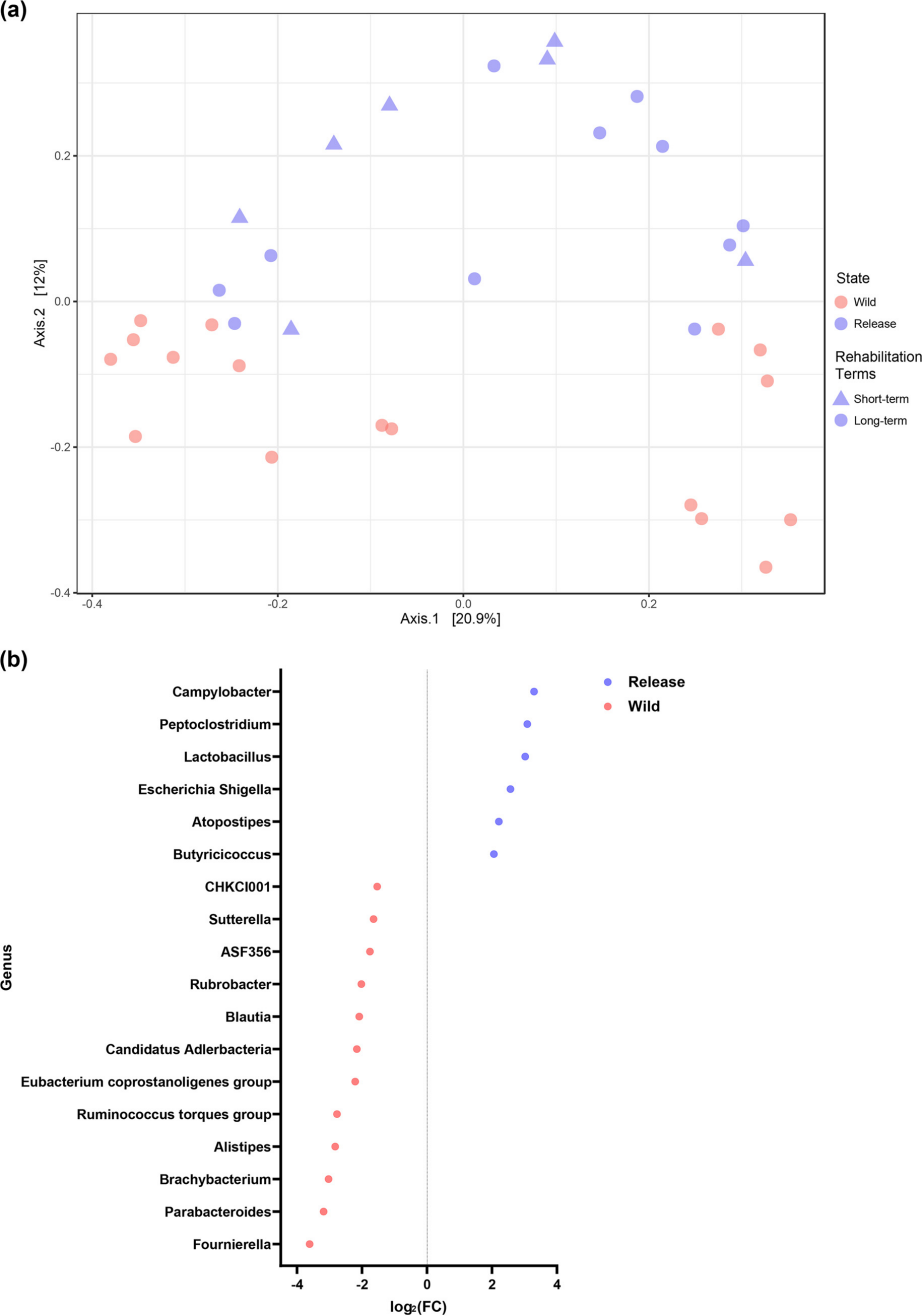
Shifts in the beta diversity of the gut microbiome of wild migratory birds during rehabilitation. (a) Principal coordinates analysis based on unweighted UniFrac distance. Birds in the wild and release states are clustered in different sections of the PcoA plot. (b) Differential abundance analysis of the gut microbiome of birds in the wild and release states.

Shifts in beta diversity were evaluated using principal coordinate analysis (PcoA) based on the unweighted UniFrac distance, followed by permutational multivariate analysis of variance (PERMANOVA). There was a significant difference in the gut microbiome composition between the wild and release states (*P < *0.05). Moreover, samples taken from wild birds immediately before their release were clustered, regardless of the period of rehabilitation (Fig. 4a).

To explore the specific components of the gut microbiome that contributed to this shift, and thus to the disruption of the microbiome composition and function (dysbiosis), we performed differential abundance analysis between the wild and release states. *Proteobacteria* was the only phylum significantly enriched in the release state compared to the wild state (adjusted *P < *0.05). Twelve genera (Fig. 4b), namely, *Brachybacterium*, *Alistipes*, *Fournierella*, *Parabacteroides*, the Ruminococcus torques group, the Eubacterium coprostanoligenes group, *CHCKI001*, *Blautia*, *Sutterella*, *Rubrobacter*, *Reyranella*, and *ASF356*, were significantly decreased in the release state compared to the wild state (adjusted *P < *0.05). In contrast, only five genera (*Atopostipes*, Escherichia*-Shigella*, Campylobacter, *Lactobacillus*, and *Peptoclostridium*) were significantly enriched in the release state compared to the wild state (adjusted *P < *0.05).

### Shifts in gut microbiome ecological interactions during rehabilitation.

**FIG 5:**
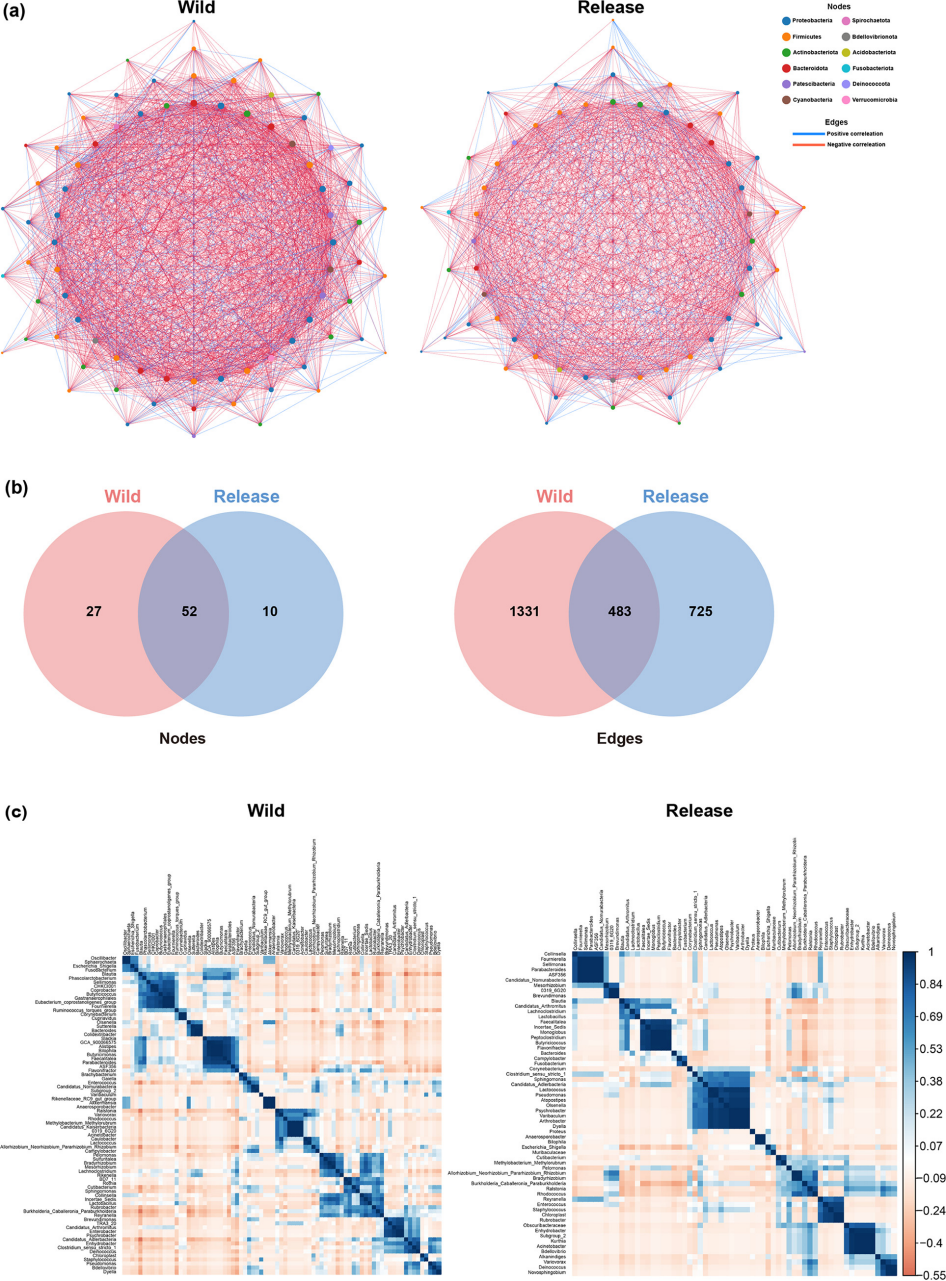
Shifts in the gut microbiome ecological interactions due to rehabilitation. (a) Co-occurrence networks of the gut microbiome in the wild and release states at the genus level. Networks were constructed using NAMAP with Pearson’s correlation. Statistically significant associations using *P < *0.05 and *r *> 0.7 as cutoff values and 100 bootstrapping iterations are shown. The colors of nodes indicate the phylum each genus belongs to, and the sizes of the nodes represent their degree (number of edges). Blue lines indicate a positive correlation and red lines indicate a negative correlation. (b) Venn diagrams show the shared and unique nodes and edges of the co-occurrence networks in the wild and release states. (c) Corresponding correlograms for the networks of the gut microbiome in the wild (left) and release (right) states.

To elucidate the shifts in the ecological interactions among gut microorganisms during rehabilitation, co-occurrence networks were constructed for the wild and release states. Seventy-nine and 62 genera (nodes) were considered significant (*P < *0.05, *r *> 0.7) in the wild and release state networks, respectively (Fig. 5a). Moreover, 1,814 correlations (edges) were observed in the wild state network, whereas 1,208 edges were observed in the release state network. The networks of the gut microbiome in the wild and release states shared 52 nodes and 483 edges (Fig. 5b). The numbers of unique edges and nodes in the network of the gut microbiome in the wild state were 27 and 1,331, respectively, while the network of the gut microbiome in the release state comprised 10 unique edges and 725 unique nodes. Compared to the wild state, ecological interactions between the gut microorganisms were attenuated in the release state, as shown by the decrease in average degree values from 45.35 to 38.35. The corresponding correlograms for the networks of the gut microbiome in the wild and release states are shown in Fig. 5c. Detailed information on the nodes and edges is provided in Tables S2 and S3 in the supplemental material, respectively.

### Shifts in the gut microbiome metabolic pathways during rehabilitation.

**FIG 6:**
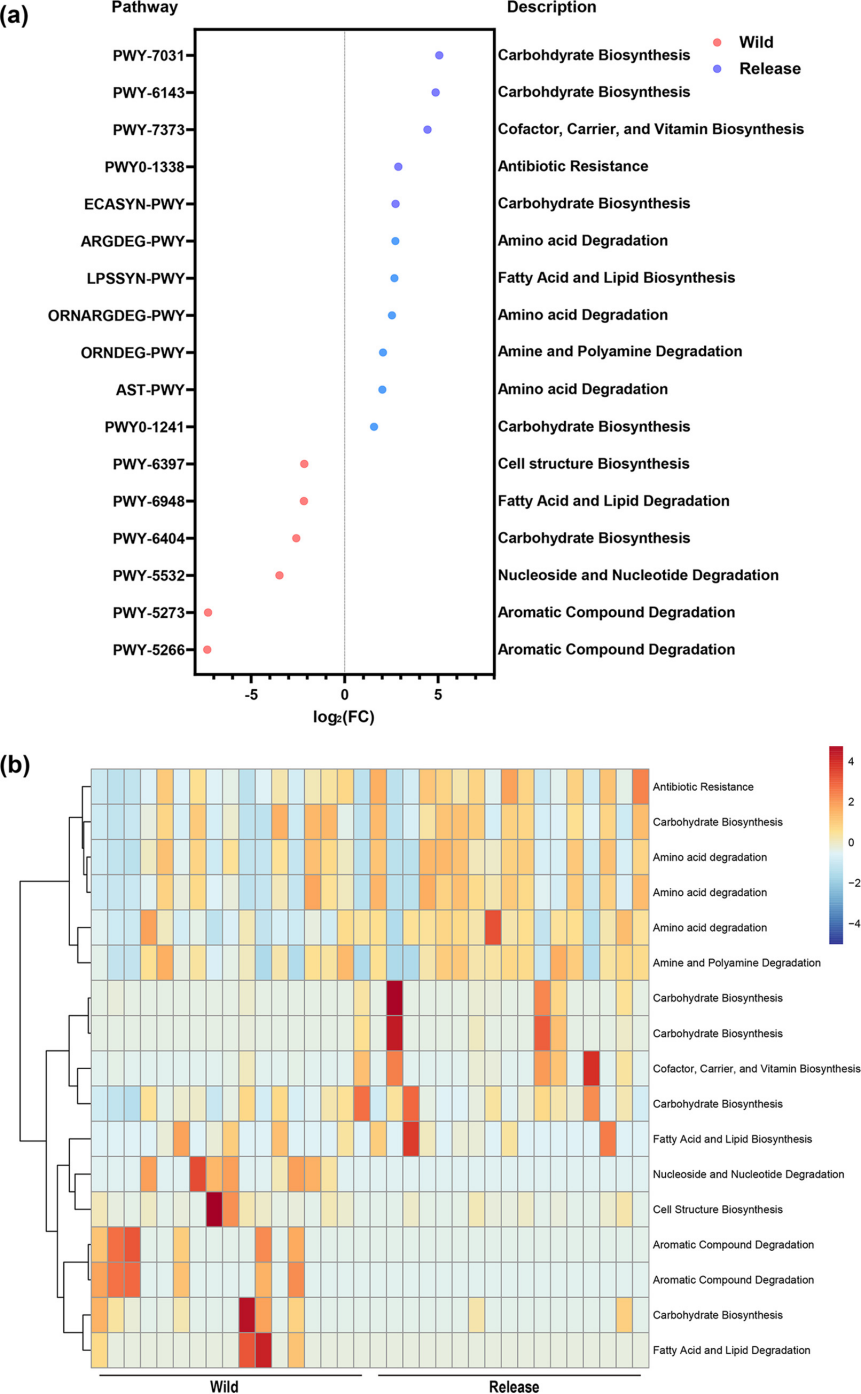
Shifts in the gut microbiome metabolic pathways due to rehabilitation. (a) Differential abundance analysis of potential metabolic pathways in the wild and release states. (b) Heatmap of metabolic pathways differing significantly between the wild and release states.

The impact of rehabilitation on the metabolic pathways of the gut microbiome was analyzed using PICURSt2 software (Fig. 6a and b). Differential abundance analysis showed that six metabolic pathways, including aromatic compound degradation, nucleoside and nucleotide degradation, glycan biosynthesis, fatty acid and lipid degradation, and cell structure biosynthesis, were significantly enriched (adjusted *P < *0.05) in the wild state. On the other hand, 11 metabolic pathways, including carbohydrate biosynthesis, cofactor, carrier, and vitamin biosynthesis, lipopolysaccharide biosynthesis, glycan biosynthesis, amino acid degradation, fatty acid and lipid biosynthesis, amine and polyamine degradation, and carbohydrate biosynthesis, were significantly enriched (adjusted *P < *0.05) in the release state.

### Wild birds acquire antibiotic resistance during rehabilitation.

**FIG 7:**
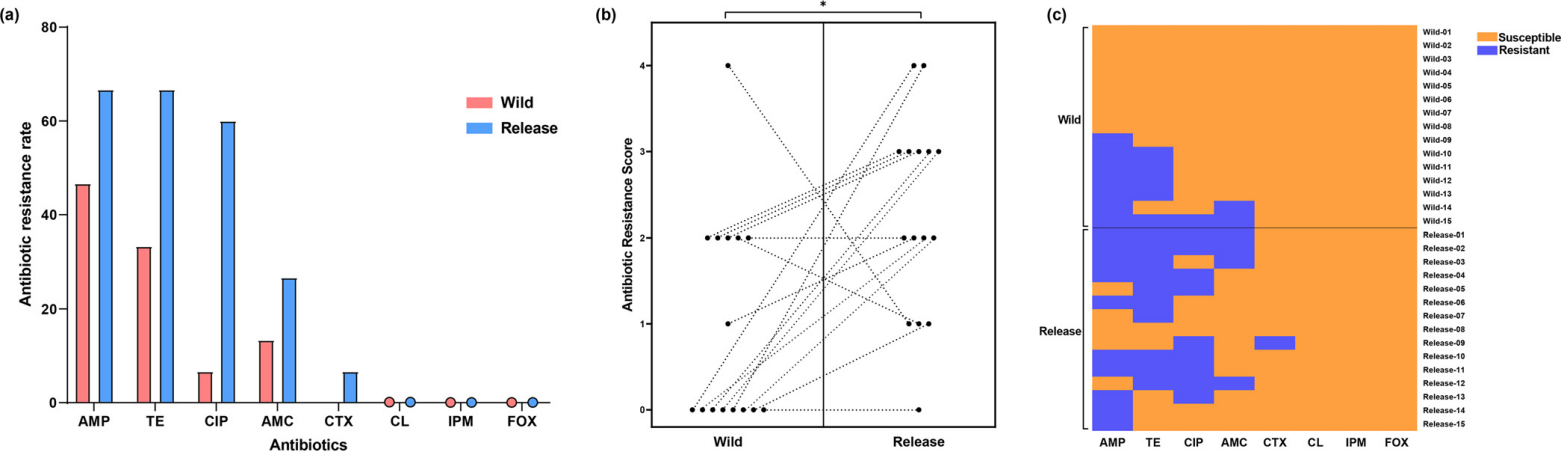
Shifts in antibiotic resistance owing to rehabilitation. (a) Antibiotic resistance rates to the eight types of antibiotics used in the present study. (b) Dot plots show the paired sample analysis of antibiotic resistance scores in the wild and release states. (c) Heatmap of the antibiotic resistance in the wild and release states.

To explore if antibiotic resistance of the gut microbiome of wild birds shifted during rehabilitation, we isolated Escherichia coli from fecal samples. The 30 E. coli strains isolated from 15 of the 17 wild birds (one isolate each per bird for the wild and release states) were then tested for antibiotic susceptibility. In the wild state, E. coli showed the highest resistance rate to ampicillin (46.67%), followed by tetracycline (33.33%), amoxicillin (13.33%), and ciprofloxacin (6.67%). In the release state, E. coli showed the highest resistance rate to ampicillin and tetracycline (both at 66.66%), followed by ciprofloxacin (60.00%), amoxicillin (26.67%), and cefotaxime (6.67%). E. coli showed no resistance to colistin, imipenem, and cefoxitin in both the wild and release states (Fig. 7a). Antibiotic-resistant scores, determined by the number of antibiotic resistance phenotypes, were significantly increased (Wilcoxon test, *P < *0.05) in the release state compared to that in the wild state (Fig. 7b). Notably, seven (87.5%) of the eight E. coli strains with no antibiotic resistance in the wild state acquired multidrug resistance in the release state (Fig. 7c).

## DISCUSSION

As the host gut microbiome is closely associated with host fitness, it is essential to better understand the influence of wildlife rehabilitation procedures on the dynamics of the gut microbiome to establish sustainable rehabilitation strategies. The present study aimed to explore the impact of rehabilitation on the gut microbiome of wild migratory birds, as it may affect their adaptability after being released into natural habitats. We hypothesized that environmental stress during rehabilitation may induce gut microbiome dysbiosis and increase antibiotic resistance in wild migratory birds. Therefore, we investigated the dynamics of the gut microbiome of wild migratory birds using longitudinal sampling, 16S rRNA sequencing, and antibiotic resistance monitoring.

In the present study, the overall taxonomic composition of the gut microbiome of birds in the wild and release states differed at both the phylum and genus levels. The phylum *Firmicutes*, which is involved in the metabolism of carbohydrates, polysaccharides, and fatty acids (9, 25), was the most dominant in the wild state, in agreement with previous findings (16, 26). However, during rehabilitation, the phylum *Proteobacteria* increased significantly, and it constituted a dominant proportion of the gut microbiome of wild birds in the release state. Indeed, a high abundance of *Proteobacteria* is an indicator of gut microbiome dysbiosis and epithelial dysfunction (27). Our results, therefore, support that rehabilitation can lead to gut microbiome dysbiosis.

In our study, the dynamics of alpha diversity demonstrated rapid and irreversible dysbiosis during rehabilitation. This dysbiosis of the gut microbiome during rehabilitation may be due to alterations in the diet and/or habitat of the wild birds at the rehabilitation center. The wild bird species investigated in the present study primarily hunt and feed on different vertebrates and invertebrates, ranging from insects to large animals, and thereby have a highly diverse diet (28). However, during rehabilitation, they were only fed chicks, which is far from representing their diets in the wild. Dietary modifications in rehabilitation centers excluding the diverse components from the wild environment may therefore deplete certain microorganisms by purging necessary nutrients, resulting in decreased species richness of the gut microbiome. Previous studies have reported that diet alteration induces a rapid shift in the composition and diversity of the gut microbiome, which is not recoverable even after the reintroduction of the original diet (29–31). In the present study, wild birds were kept in cages at the rehabilitation center, and so the conditions differed from those in their wild habitat. This may have contributed to the shift of the gut microbiome during rehabilitation, as birds in their wild habitats are exposed to diverse microorganisms and environmental factors, all of which are involved in shaping the gut microbiome. Consistent with our results, previous studies have shown that habitat changes significantly alter the gut microbiome of wild animals (32, 33). Overall, the present study indicates that environmental stresses during rehabilitation, including alterations in diet and habitat, may have induced dysbiosis of the gut microbiome of wild migratory birds.

Notably, most of the microbes that significantly decreased after rehabilitation were short-chain fatty acid (SCFA) producers, such as *Parabacteroides* and *Blautia*. SCFAs are generated by the fermentation of carbohydrates and are essential for gut integrity and host health (34). A decrease in SCFA-producing bacteria in the gut microbiome is associated with various physiological and metabolic disorders due to the loss of gut integrity (35). Thus, our results suggest that the decrease in the SCFA-producing bacteria in the gut microbiome during rehabilitation may negatively affect the fitness of wild migratory birds after their release into the wild environment. Dietary modifications are the major factors associated with a decrease in SCFA-producing bacteria in the gut (36). For birds, insects are major dietary sources of SCFA-producing bacteria (37–39). As the birds used in the present study were only fed chicks during rehabilitation, the observed decrease in SCFA-producing bacteria may be due to the lack of diet variability, which resulted in a lack of nutrients for the growth of these bacteria.

Bacteria that were enriched in the release state were mainly zoonotic pathogens, such as Campylobacter and *Peptoclostridium*, indicating that pathogenic species were able to colonize the guts of wild migratory birds during rehabilitation. If an external infection was the source of this increase in zoonotic pathogens, clinical signs, such as lethargy, diarrhea, or behavioral changes, would have been observed; however, none of the birds in the present study showed obvious signs of infection. Considering the absence of clinical signs and the results of the 16S rRNA sequencing analysis, these pathogens may have colonized the gut of wild migratory birds during rehabilitation as common members of the gut microbiome. Our result is consistent with that of a previous study, which showed that several zoonotic pathogens, such as Campylobacter, increased in the gut microbiome of wild animals during rehabilitation (21). This may be due to (i) a decrease in SCFA-producing bacteria and/or (ii) a modified diet during rehabilitation. SCFA-producing bacteria are known to inhibit colonization by pathogens, such as Campylobacter and *Peptoclostridium*, by directly regulating microorganism-microorganism interactions and indirectly regulating host-microorganism interactions (40, 41). Thus, the decrease in SCFA producers may have played a key role in the overgrowth of zoonotic pathogens in the guts of wild migratory birds. Moreover, chicks and quail commonly harbor Campylobacter and *Peptoclostridium* in their gut microbiome (42, 43). As wild birds in our study were fed whole carcasses of chicks and quail during rehabilitation, the components of the gut microbiomes of these species, including Campylobacter and *Peptoclostridium*, may have been transmitted to the wild migratory birds. The enrichment of zoonotic pathogens in the gut microbiomes of wild birds after rehabilitation may increase their potential to be transmitted to humans and other animals, with migratory birds serving as the link between wildlife and human communities after they are released into the wild.

The present study demonstrated that the complexity of microbial interactions, which can be inferred by the number of nodes and edges, decreased during rehabilitation. This may be due to the decreased species richness induced by rehabilitation, as shown by the alpha and beta diversity analyses of the gut microbiome. Because several species were depleted, several nodes with significant correlations were lost. A previous study showed that environmental stress reduces the complexity of the microbiome network, supporting our findings (44). Notably, our study showed that the unique nodes observed in the wild state mainly included SCFA producers. This is consistent with our differential abundance analysis results, which showed that SCFA producers were depleted during rehabilitation, along with the complex microorganism-microorganism interactions they mediate. As microbial interactions mediated by SCFA producers are associated with a wide range of host fitness factors, including immune and metabolic functions (45, 46), this depletion of the microbiome network may negatively affect wild birds after their release.

The metabolic pathways that were the most affected during rehabilitation were those involved in the degradation of aromatic compounds. Aromatic compounds are the most widespread and abundant pollutants in the natural environment and diet of wild birds (47, 48). Wild birds, particularly birds of prey, have the potential to accumulate high concentrations of aromatic compounds in wild environments and therefore harbor microorganisms that are able to biodegrade these compounds in their gut (49–51). Thus, the enriched metabolic pathways involved in aromatic compound degradation in the wild state may reflect a strategy used by wild migratory birds to survive in wild environments. However, our results showed that rehabilitation downregulated the metabolic pathways involved in aromatic compound degradation, which may be due to the alterations in diet and habitat that shifted the gut microbiome diversity and composition. These results indicate that the fitness of wild migratory birds to degrade aromatic pollutants and aromatic compound-rich diets might be decreased when these birds are released into their natural habitats. Collectively, this rehabilitation-induced shift in the metabolic pathways of the gut microbiome may affect the adaptation to wild migratory environments after release.

Wild birds are known as potentially important sources of antibiotic resistance dissemination in the environment (19). E. coli is a well-established antibiotic resistance indicator for evaluating the anthropogenic impact on the environment (52). Therefore, we isolated E. coli from fecal samples of wild birds and examined the shift in its antibiotic resistance during rehabilitation. Notably, our results showed that antibiotic resistance was significantly increased during rehabilitation. Since none of the birds used in the present study was treated with antibiotics during rehabilitation, the increased antibiotic resistance observed is unlikely to have resulted from antibiotic exposure. Antibiotics associated with increased resistance included ciprofloxacin, ampicillin, amoxicillin, and tetracycline, which are the most frequently used drugs in veterinary clinics (53). In a clinical environment with frequent exposure to these antibiotics, environmental microbes acquire antibiotic resistance and opportunistically infect hosts (54, 55). Therefore, it can be inferred that the microorganisms of wild birds acquired antibiotic resistance from the rehabilitation environment via the colonization of antibiotic-resistant environmental microorganisms. However, wild birds did not acquire resistance to colistin and imipenem, which are drugs of last resort and are thus rarely used in the clinical environment (56, 57). Environmental microorganisms in the rehabilitation environment were, therefore, less exposed to colistin and imipenem, and the microorganisms of wild birds did not acquire resistance to these antibiotics.

Dietary modifications during rehabilitation may also have contributed to the acquisition of antibiotic resistance in wild birds. Indeed, 1-day-old chicks are a major source of in-farm-transmitted antibiotic resistance owing to their high antibiotic resistance levels (58, 59). The remaining antibiotic-resistant microorganisms in the guts of chicks could have colonized the guts of wild birds during rehabilitation, resulting in increased antibiotic resistance. Collectively, our findings indicate that wild birds may acquire antibiotic resistance during rehabilitation and thus serve as the source of antibiotic resistance spread in the environment after they are released.

### Conclusions.

Our study shows that wildlife rehabilitation induces alterations in the gut microbiome and the acquisition of antibiotic resistance in wild migratory birds. The diversity of the gut microbiome significantly decreased during rehabilitation, and it did not recover to the level observed in the wild state, indicating the possibility of rehabilitation-induced dysbiosis. Moreover, zoonotic pathogens, including *Peptoclostridium* and Campylobacter, were enriched, whereas SCFA-producing bacteria were depleted in the gut of wild migratory birds at the end of the rehabilitation period, and the ecological network of the gut microbiome showed decreased complexity. Metabolic pathways involved in the degradation of aromatic compounds were significantly downregulated, indicating that the ability of the gut microbiome to degrade these pollutants might have been compromised. Overall, these results indicate that the rehabilitation-induced dysbiosis of the gut microbiome of wild migratory birds may affect their adaptation to the wild environment after release. Moreover, wild birds may serve as a potential source of the dissemination of antibiotic resistance when released into wild environments because they may acquire antibiotic resistance during rehabilitation. Therefore, more attention should be devoted to studying the dynamics of the gut microbiome of wild migratory birds during rehabilitation, in order to achieve sustainable rehabilitation strategies.

## MATERIALS AND METHODS

### Rehabilitation and gut microbiome sampling of wild migratory birds.

Orphaned wild migratory birds were rescued in Seoul, South Korea, and immediately transferred to the Seoul Wildlife Rehabilitation Center. They were kept in individual cages, following the rehabilitation manual of the Seoul Wildlife Center. One-day-old chicks (sourced from the poultry industry) and captive-bred quail were provided to birds that were alert and had the ability to feed themselves. The health status and behavior of the rescued wild birds were monitored daily by veterinary staff at the Seoul Wildlife Center. Injured and convalescent wild birds, birds showing abnormal clinical signs, and juvenile birds not capable of self-feeding were excluded from the study. Thus, all birds used in the present study were clinically healthy. When birds were physically and behaviorally able to forage and breed in the wild, and therefore considered ready for release by veterinarians, they were released into a native forest or park. Wild birds that were rehabilitated for less than 4 weeks were considered to have undergone short-term rehabilitation, while those that were rehabilitated for 4 weeks or more were considered to have undergone long-term rehabilitation. Information regarding the wild birds used in the present study is provided in Table S1 in the supplemental material.

Fecal samples of the wild birds were collected by veterinarians, immediately transferred to the laboratory, and processed for gut microbiome 16S rRNA sequencing analysis and antibiotic resistance monitoring. Sample collection was performed in the wild state, within the first 2 weeks of rehabilitation, and then at 4-week intervals until release. Fifty fecal samples were used for DNA extraction and microbiome 16S rRNA sequencing analysis (Fig.1b).

### DNA extraction, library preparation, and 16S rRNA sequencing.

Fecal DNA extraction and sequencing were performed as previously described (14). Briefly, DNA was extracted from fecal samples using a Fast DNA soil kit (MP Biomedicals, Santa Ana, CA, USA) following the manufacturer’s instructions. Sequencing of the *16S rRNA* V3-V4 hypervariable gene region was performed using the primers 341F (5′-TCGTCGGCAGCGTCAGATGTGTATAAGAGACAGCCTACGGGNGGCWGCAG-3′) and 805R (5′-GTCTCGTGGGCTCGGAGATGTGTATAAGAGACAGGACTACHVGGGTATCTAATCC-3′) from Illumina Inc. (San Diego, CA, USA). PicoGreen was used for pooling and normalizing the amplified products. All sequencing procedures were conducted using the Illumina MiSeq platform at Macrogen, Inc. (Seoul, South Korea).

### Bioinformatics and statistical analyses.

Bioinformatics analysis of the sequence data was performed using the QIIME2 (version 2021.02) software package (60). Raw sequence data were filtered, dereplicated, and denoised to generate ASV tables, using DADA2 as implemented in QIIME2 (61). A phylogenetic tree of the ASVs was generated using MAFFT (https://mafft.cbrc.jp/alignment/software/). The taxonomy profile of ASVs was generated using the q2-feature-classifier implemented in QIIME2 against the SILVA database (version 138, Ref NR99) (62). Sequence data were normalized using the rarefaction to the minimum library size method for downstream analysis.

Downstream analysis of sequence data was performed using the MicrobiomeAnalyst R package (63). The alpha diversity of the microbiome was measured using the number of observed ASVs and Shannon’s index. The significance of differences in alpha diversity was evaluated using the Mann-Whitney U test for intergroup comparisons and the Wilcoxon test for paired samples. The beta diversity of the microbiome was measured using the unweighted UniFrac distance, followed by PERMANOVA to evaluate significant differences in beta diversity. Differential abundance analysis of the gut microbiome and its metabolic pathways was performed using edgeR (64). A co-occurrence network was constructed using network analysis for metagenomic abundance profiles (NAMAP) based on Pearson’s correlations, using the MetagenoNets tool (65, 66) with *r* > 0.7 and *P* < 0.05 as the cutoff values for significant correlations. Metabolic pathways were analyzed using the phylogenetic investigation of communities by reconstruction of unobserved states (PICRUSt2) (67) and the MetaCyc database (https://metacyc.org).

### Isolation of Escherichia coli for antibiotic susceptibility tests.

To isolate E. coli for monitoring antibiotic resistance in wild birds during rehabilitation, fecal swabs from 17 wild birds in the wild state and release state were inoculated into 2 mL of E. coli broth (Oxoid, Basingstoke, UK) and enriched overnight at 37°C. After enrichment, 100 μL of the culture broth was spread on MacConkey agar (Oxoid) and incubated at 37°C for 24 h. Cultures were then streaked on eosin methylene blue agar (BD, Sparks, MD, USA), and colonies exhibiting the culture characteristics of E. coli were pure cultured and confirmed by matrix-assisted laser desorption ionization–time of flight mass spectrometry. As a result, E. coli was isolated from 15 birds and further analyzed for antibiotic susceptibility.

Disk diffusion susceptibility tests (Kirby-Bauer method) were conducted for eight antibiotics: amoxicillin-clavulanic acid (20/10 μg), ampicillin (10 μg), cefotaxime (30 μg), imipenem (10 μg), tetracycline (30 μg), ciprofloxacin (5 μg), colistin (10 μg), and cefoxitin (30 μg). Antibiotic susceptibility results were interpreted following the Clinical and Laboratory Standards Institute guidelines.

### Ethics declarations.

The rehabilitation program at the Seoul Wildlife Center was conducted with the authorization of the Seoul Metropolitan Government and Ministry of Environment of Korea. All procedures in this study strictly followed the guidelines and ethical principles of the following South Korean legislation: the Wildlife Protection and Management Act, the Guidelines for the Operation of the Wildlife Rescue and Management Center by the Ministry of Environment of Korea, and the Cultural Heritage Protection Act by the Cultural Heritage Administration of Korea.

### Data availability.

Sequence data from this study are deposited in the National Center for Biotechnology Information Short Read Archive database under accession number PRJNA814404.
